# The effect of neural pre-stimulus oscillations on post-stimulus auditory ERPs in disorders of consciousness

**DOI:** 10.3389/fnins.2025.1547167

**Published:** 2025-04-08

**Authors:** Laura Lindenbaum, Inga Steppacher, Alexandra Mehlmann, Johanna Maria Kissler

**Affiliations:** ^1^Department of Psychology, Bielefeld University, Bielefeld, Germany; ^2^Center for Cognitive Interaction Technology (CITEC), Bielefeld University, Bielefeld, Germany

**Keywords:** disorder of consciousness (DOC), minimally conscious state (MCS), unresponsive wakefulness syndrome (UWS), EEG frequency bands, EEG, auditory event related potentials

## Abstract

**Objective:**

Pre-stimulus oscillations predispose subsequent stimulus detection, but the connection between the pre-stimulus EEG activity and post-stimulus event-related potentials (ERPs) has rarely been examined in people in a disorder of consciousness (DoC). Hence, we investigate how pre-stimulus EEG band power is related to post-stimulus ERPs in individual DoC patients.

**Methods:**

We conducted an active auditory oddball paradigm encompassing standard, target and unexpected oddball stimuli with 14 DoC patients (*N* = 12 minimally conscious state [MCS], *N* = 2 unresponsive wakefulness syndrome [UWS]). We extracted post-stimulus ERPs as well as pre-stimulus power-spectra.

**Results:**

P3-like differences between brain responses to auditory stimuli were found in seven patients (50%). Delta and theta bands pre-dominated in all patients’ pre-stimulus frequency spectra but patients with significant post-stimulus P3 had on average more pre-stimulus beta and gamma power than those without P3 effects. Pre-stimulus power and post-stimulus ERPs correlated in five patients (36%). Several patients showed negative correlations between pre-stimulus gamma and beta power and post-stimulus ERP variables, suggesting a u-shaped relationship between pre-stimulus high-frequency activity and post-stimulus ERP. Only one patient showed a relationship between pre-stimulus alpha and ERP as previously found in healthy people.

**Conclusion:**

Pre-stimulus frequencies in DoC were related to post-stimulus processing at least in some patients. The pattern of the relationship showed considerable variability underscoring substantial alterations in brain activity among patients with DoC. The comparison with somatosensory results in the same patients emphasizes the need for multi-modal assessment.

**Significance:**

The high inter-individual variability in the connection between pre-stimulus oscillations and auditory processing in DoC necessitates extensive individual assessment to determine optimal stimulation windows for DoC patients.

## 1 Introduction

Traumatic and non-traumatic brain injuries can lead to various adverse consequences, in the most severe cases including Disorders of Consciousness (DoC). DoC can be categorized into distinct stages like the Minimally Conscious State (MCS) (Giacino et al., 2002) and the Unresponsive Wakefulness Syndrome (UWS), previously referred to as the Vegetative State (VS) (Laureys et al., 2010). These stages are primarily classified based on observable behavioral characteristics and their inferred connection to the individual’s level of consciousness. UWS-patients exhibit no indications of self-awareness or environmental awareness (Jennett and Plum, 1972) and MCS-patients exhibit slight yet repeatable signs of self-awareness and/or awareness of their surroundings (Giacino et al., 2002, 2014).

MCS can be further sub-divided into MCS-and MCS+ with MCS+ diagnosed for patients with high level responses (e.g., command following, gestural or verbal yes/no responses) and MCS-for patients with low-level responses (e.g., localization of noxious stimuli, pursuit eye movements that occur in direct response to moving or salient stimuli) (Bruno et al., 2011). MCS and UWS can persist as permanent states without any discernible changes in the patient’s cognitive status for many years, ultimately leading to death but there is also potential for improvement and recovery of the patient. Patients in MCS and those who attain MCS more rapidly following injury and coma may seem more likely to experience such progress (Giacino and Kalmar, 1997, 2005; Steppacher et al., 2016, 2020).

The brain activity of people who suffer from a DoC is diffuse and highly different from healthy people. To enhance our understanding of the cognitive functions and neural processes of patients with DoC, neuroscience research has employed various of methodologies, such as electroencephalography (EEG), functional magnetic resonance imaging (fMRI), positron emission tomography (PET), and magnetoencephalography (MEG). These investigations demonstrate that on occasion, patients who receive a behavioral diagnosis of UWS exhibit brain activity that suggests the presence of advanced cognitive processes, resembling patterns observed in healthy individuals (e.g., Owen et al., 2006; Cruse et al., 2011) and that certain individuals within the UWS and MCS are also able to respond to commands and modulate their brain activity to perform a cognitive task. But these findings are variable between individuals since disease duration and traumatic pathology are also related to reliably detectable brain responses (Kotchoubey et al., 2005). Additionally, misdiagnosis in patients with DoC is alarmingly common, with many being incorrectly classified as UWS instead of MCS based on single clinical assessments, an error that can be reduced with repeated (at least five) assessments (Wannez et al., 2017). Also, to help prevent misdiagnosis, auditory localization, i.e., the ability to behaviorally orient towards an auditory stimulus appears useful to differentiate between MCS and UWS. Multimodal brain imaging findings indicate more efficient brain functioning in patients with auditory localization, supporting the idea that auditory localization should be considered as a sign of MCS (Carrière et al., 2020). Given the limitations of fMRI, PET, and MEG in clinical applications, EEG presents a practical alternative for bedside examinations and the assessment of consciousness. This is due to the fact that ERPs (Event-Related Potentials) and oscillations in EEG recordings have been proposed as significant indicators of consciousness, with the potential to predict the prognosis of patients with DoC (Chennu et al., 2017; Fischer et al., 2004; Kotchoubey, 2005; Luauté et al., 2005; Steppacher et al., 2020). Important ERPs are for example the N100 and the P300, which can be identified by their polarity and latency in the pre-processed and averaged EEG-Data (Squires et al., 1975; Lindenbaum et al., 2021). The N100 is a negative deflection that occurs around 100 ms after stimulus onset and corresponds to the sensory processing of the stimuli (Näätänen and Picton, 1987). In contrast, the P300, a positive deflection after approximately 300 ms which occurs in discrimination or oddball tasks, reflects the detection of differences along some specific dimension of the stimuli properties (Polich, 1989) and is a response to target deviant or non-target deviant stimuli. The latter are, referred to as novel stimuli. Whereas deviant targets repeat themselves, albeit at a low frequency, and require a specific response such as button press or counting of their occurrences, task-irrelevant novel stimuli do not repeat and do not require a response from the subject. Novels typically elicit an earlier fronto-central P300, the novelty P300 (nP3) or P3a, whereas the target deviants elicit a later parietal P300, the P3b. ERPs, such as the P300, have also been consistently identified as reliable indicators of an individual’s current level of consciousness (Engemann et al., 2018; Kotchoubey, 2005). Likewise, EEG oscillations have been shown to serve as significant indicators of consciousness and awareness (Balconi, 2011). They are linked to different states of consciousness and cognitive processes, and may serve as potential indicators of the neural mechanisms underlying conscious experiences. These oscillations can be categorized into distinct frequency bands including delta (<4 Hz), theta (4–8 Hz), alpha (8–12 Hz), beta (12–30 Hz), and gamma (>30 Hz). The alpha frequency band plays an active role in inhibitory processes, attentional modulation and memory functions during cognitive information processing reflecting anticipatory and temporal attention (Klimesch, 2012). Alpha is also the predominant oscillation observed in the EEG of healthy adults (Klimesch, 1999).

In patients with disorders of consciousness, the relative strength or power of these frequency bands often shows significant abnormalities. Typically, there is a decrease in alpha band power and an increase in delta band power. Specifically, relative delta band power tends to be higher in patients with unresponsive wakefulness syndrome compared to those with minimally conscious state, while alpha and theta power is higher in MCS patients when compared to those with UWS (Lehembre et al., 2012b; Sitt et al., 2014; Piarulli et al., 2016). Also, a consistent presence of reduced alpha-theta power coupled with increased delta activity is a reliable indicator of unconsciousness (Sitt et al., 2014).

Not even in healthy people is the responsiveness to stimulation stable at all times. Instead, the oscillatory activity before stimulus-onset in cognitive tasks has been demonstrated to influence subsequent conscious auditory perception (Henry and Obleser, 2012; Henry et al., 2016; Ng et al., 2012). Particularly, the power and phase of the alpha-frequency band play a significant role in this influence. Numerous studies have indicated that, in healthy people, pre-stimulus oscillations are related to post-stimulus ERPs, especially regarding the P300 component (Ergenoglu et al., 2004; Haig and Gordon, 1998; Jasiukaitis and Hakerem, 1988; Mathewson et al., 2009) and N100 component (Intriligator and Polich, 1995; Roberts et al., 2014). Lower pre-stimulus relative alpha power is followed by significantly higher post-stimulus P300 responses, whereas high pre-stimulus alpha results in only small ERPs at least in the visual paradigm (Ergenoglu et al., 2004). In the auditory paradigm, Jasiukaitis and Hakerem (1988) showed a positive relationship between pre-stimulus spectral power in the alpha band and the amplitude of the P300. Haig and Gordon (1998) demonstrated in an auditory oddball paradigm that the P300 is affected by the pre-stimulus alpha phase and that some pre-stimulus alpha phases are associated with the presence or absence of a large P300. Also, Pritchard et al. (1985) examined the relationship between pre-stimulus EEG power and the amplitude of post-stimulus P300 in a visual paradigm. They found positive correlations between the pre-stimulus alpha power and post-stimulus P300 and a negative correlation between the pre-stimulus delta power and post-stimulus P300.

Most studies examined the relationship between pre-stimulus alpha frequency power and post-stimulus ERPs but also correlations between other pre-stimulus frequency bands and post-stimulus ERPs have been found. The results of Reinhart et al. (2011) showed a positive correlation between pre-stimulus gamma power and post-stimulus P300 amplitudes in healthy people. Post-stimulus P300 amplitudes are more positive when the pre-stimulus gamma power is higher. De Blasio and Barry (2013a) showed a positive correlation between pre-stimulus delta activity and post-stimulus P300 amplitudes. Higher pre-stimulus delta activity was associated with more positive post-stimulus amplitudes. Other studies reported positive correlations between pre-stimulus theta activity and post-stimulus P300 (Başar et al., 1984; De Blasio and Barry, 2013a; Yordanova and Kolev, 1998; Lazzaro et al., 2001). But so far, these studies have largely been conducted with healthy volunteers. In our previous study we examined the relationship between pre-stimulus oscillations and post-stimulus variables to a somatosensory paradigm in patients with DoC (Lindenbaum et al., 2023). We found statistically significant correlations between pre-stimulus power and post-stimulus event-related brain responses in five out of 14 patients. Comparable correlation patterns to those found in healthy individuals were predominantly observed between relative pre-stimulus alpha power and post-stimulus variables during later time-intervals, although contrasting effects were also observed.

In the current study, we investigated the connection between pre-stimulus EEG activity and post-stimulus auditory responses in individuals with a disorder of consciousness. Because patients in UWS and MCS cannot always sustain eye-opening or control their eye movements and fixate a specific point within their visual field, most ERP-studies in DoC rely on auditory paradigms. There are multiple studies investigating auditory ERPs in people with DoC (e.g., Fischer et al., 2010; Kotchoubey et al., 2005; Risetti et al., 2013) but the presence of ERPs are very variable and intra-individual fluctuations can contribute to this outcome. Hence, we conducted an analysis of pre-stimulus oscillations and post-stimulus auditory ERPs (aERPs), to be precise the N100 and P300, in response to an active three-sound auditory oddball paradigm (standard, deviant (target), novel (non-target)). Due to the diversity of brain lesions and their location in our patients, our initial analysis focused on the post-stimulus epochs of standard, deviant and novel stimuli. We aimed to identify statistically significant differences in electrode locations and time-intervals between these stimuli. Subsequently, we determined the latency of the amplitude maximum, the maximum itself and also the area under curve (AUC) of the post-stimulus aERPs within the identified significant time-interval. Additionally, the relative power of frequency bands in the pre-stimulus epochs for the deviant and novel stimuli were computed. For each patient, we conducted individual tests to evaluate whether there was a correlation between the relative power of pre-stimulus frequency bands and post-stimulus variables. This assessment aimed to determine whether pre-stimulus oscillation frequencies could predispose post-stimulus outcomes in the auditory stimulation condition.

## 2 Patients and methods

The method and data analysis used in this research rely on those applied by Lindenbaum et al. (2023), with the difference that this study focuses on an auditory paradigm.

### 2.1 Patients

Fourteen DoC-patients (6 female) living at the care facility “Haus Elim MeH,” Bethel, in Bielefeld Germany, participated in this study, which was conducted from July 2017 to August 2018. The data sample is the same as in the study of Lindenbaum et al. (2023) and consists of 12 MCS and 2 UWS patients (mean age-at measurement was 43.43 years). Patients have been assessed through the early functional abilities (EFA) scale (Heck et al., 2000). The EFA was created to evaluate functional recovery during the initial phase following a brain injury. It consists of 20 items, with the patient’s functional ability being assessed on a 5-point scale (Poulsen et al., 2018). Informed consent was obtained from legal representatives, primarily relatives, for each patient, and the research received approval from the ethics committee of the German Psychological Association. Detailed demographic data for all patients are provided in Table 1.

**Table 1:** Demographic information for all patients (same as in Lindenbaum et al., 2023).

Patient	Sex	Age at m.d.	Clinical State	Duration of illness (years)	Etiology
01	F	28	MCS+	9	HBD
02	F	66	MCS-	8	CCT / HBD
03	M	26	MCS-	9	CCT
04	M	53	MCS-	10	CCT / HBD
05	M	46	UWS/MCS	14	HBD
06	F	62	MCS-	12	HBD
07	M	34	MCS+	16	HBD
08	F	53	MCS	4	TI
09	M	37	MCS	1	HBD
10	F	41	MCS	2	ICH
11	F	41	MCS+	3	HBD
12	M	21	MCS+	2	IS
13	M	46	MCS	12	HBD
14	M	54	UWS	9 months	CCT

F, female; M, male; m.d., measurement date; MCS, minimally conscious state; UWS, unresponsive wakefulness syndrome; HBD, hypoxic brain damage; CCT, craniocerebral trauma; TI, thalamus infarct; ICH, intracerebral hemorrhage; IS, ischemic stroke.

### 2.2 Experimental paradigm and stimuli

The experimental paradigm was a three-sound auditory oddball paradigm with standard (1,000 Hz sine), deviant (1,500 Hz sine; target) and novel tones (nontarget). The duration of stimulus presentation was 100 ms and the inter-stimulus interval was 1,000 ms. One stimulation block consisted of 500 standard, 100 deviant and 100 synthetically generated novel stimuli (e.g., clicking, squeaking or slurping) presented in a randomized order with no two deviant or novel stimuli following consecutively. Patients were either seated in a wheelchair or reclined in their nursing bed, and were directed to focus on the high tones (deviant), count their occurrences and disregard the novel and standard tones. The stimulation block was presented twice to the patients.

### 2.3 EEG recording and preprocessing

Electroencephalography signals were recorded using a BioSemi system with 32 active electrodes^1^, a sampling rate of 2,048 Hz and Cz as the recording reference. The EEG-Data were pre-processed using BESA® Research 6.0. The collected data were down-sampled to 1,024 Hz, re-referenced to the average reference and artifacts were automatically rejected by the software. Epochs that exceeded the amplitude threshold (120 μV), gradient criterion of 75 μV, or the low signal thresholds of 0.01 μV were removed from the data.

### 2.4 Preprocessing for single-subject-aERP-analyses

The EEG-Data were pre-processed using a low-pass filter of 15 Hz (zero-phase) and a high-pass filter of 0.30 Hz (forward). The data was segmented into epochs from 100 ms before stimulus onset (baseline) to 800 ms after stimulus onset. All artifact-free epochs (see Table 2) were submitted to single-subject-analyses in EMEGS (Peyk et al., 2011). Due to the limited number of artifact-free EEG epochs available in patients with DoC, we included all remaining epochs after artifact rejection for further analysis. This approach was necessary to maximize the amount of usable data, as excluding additional epochs would reduce the available information, potentially limiting insights into the brain activity of individual patients.

**Table 2:** Artifact-free epochs of stimuli per subject which were submitted to single-subject-aERP-analyses.

Patient	Standard	Deviant	Novel
01	626	120	111
02	249	46	46
03	513	112	97
04	846	174	164
05	895	174	172
06	716	143	116
07	83	13	14
08	775	159	160
09	398	75	82
10	570	110	112
11	640	114	51
12	596	109	114
13	397	82	66
14	472	95	96

### 2.5 Preprocessing for FFT

Artifact-free trials corresponding to deviant and novel stimulation were segmented into epochs from 600 ms before stimulus to stimulus-onset (0 ms). To calculate the frequency components the trials were not filtered. The deviant and novel epochs used for FFT-analysis were identical to those employed in the single-subject-aERP-analyses.

### 2.6 Data analyses

#### 2.6.1 Single-subject-aERP-analyses

The single-subject-analysis involved cluster-based permutation tests conducted for each patient. For each time-point and electrode, a two-sample *t*-test comparing the cortical response to the different stimuli was calculated and compared against a distribution of 1,000 permutations and a cluster mass test (Maris and Oostenveld, 2007) as implemented in EMEGS (Peyk et al., 2011). The cluster mass tests were conducted one-sided testing for positive effects (e.g., deviant > standard). This process was carried out for two distinct pre-defined time intervals, during which attentional modulation of aERPs is typically anticipated (N100: 0–250 ms and P300: 250–750 ms) using all electrodes and for three different comparisons (standard vs. deviant, standard vs. novel and deviant vs. novel). The t-values obtained from the *t*-test were utilized to perform a cluster mass test, employing a significance criterion of *p* < 0.05. The significant time intervals and channel groups were identified and saved for subsequent analyses.

The epochs of standard stimuli were averaged for every patient and subtracted from every single-trial deviant and novel epoch. The difference epochs were subsequently averaged across significant channel groups within their corresponding significant time intervals. Using in-house python-based software, the most negative peak (minimum) and its latency within the N100 time-interval, as well as the most positive peak (maximum) and its latency corresponding to the post-stimulus P300 within the 250–750 ms time interval, were identified in the significant averaged difference epochs and the areas under the aERP curves (AUC) were calculated (Lindenbaum et al., 2023).

#### 2.6.2 FFT

The unfiltered single-trials of pre-stimulus deviant-and novel-epochs were averaged across the significant electrode clusters identified from the post-stimulus single-subject-analysis.

Thereafter, the averaged data were filtered using a 6th order 100 Hz digital butterworth low-pass filter, with the cut-off frequency normalized by dividing by half of the sampling frequency (Nyquist-frequency) (Lindenbaum et al., 2023). To filter out AC line noise a 50 Hz notch filter was also applied. After that, the filtered data epochs underwent windowing using a Hanning taper and additionally zero padded symmetrically at both ends to 1,024 timepoints giving 1 s of data (Lindenbaum et al., 2023). To compute the relative power of frequency components the one-dimensional discrete Fourier Transformation for real input was calculated utilizing the Python library NumPy (Lindenbaum et al., 2023). The frequency bands and their defined spectral boundaries are outlined in Table 3.

**Table 3:** Frequency bands and the defined spectral boundaries.

Frequency band	Spectral boundaries
Delta	0–4 Hz
Theta	>4–8 Hz
Alpha	>8–12 Hz
Beta	>12–30 Hz
Gamma	>30–100 Hz

#### 2.6.3 Statistical analysis

To statistically analyze the relationship between the relative pre-stimulus frequency components and post-stimulus ERP parameters, a correlation matrix was calculated separately for each subject and time interval (Lindenbaum et al., 2023). To this end, the relative power of frequency bands in the pre-stimulus interval, the maximum and minimum and latencies of the post-stimulus difference amplitudes, and the AUC of the post-stimulus significant time interval for every deviant and novel trial were used for the analysis. Correlation coefficients are reported as Spearman’s rho. With the calculation of 15 correlations for each subject and time interval, it is anticipated that some of these might be correlations occurring by chance.

#### 2.6.4 Additional analyses

In addition to the main analyses, we tested if the pre-stimulus frequency content for significant and insignificant post-stimulus intervals were statistically different. A Mann–Whitney-*U*-Test was calculated comparing the pre-stimulus interval of the deviant/novel for each of the 14 test subjects, i.e., 28 data sets (mean value of the relative frequencies in the pre-stimulus interval). This was done with a predefined centrally located P300-electrode-cluster.

We also compiled a visual comparison of the statistically significant findings of our previous somatosensory oddball study (Lindenbaum et al., 2023), carried out with the same patients as used in this study, and the significant findings of the present study (Table 4) to explore modality specific effects.

**Table 4:** Overview of significant (marked as tick) and insignificant (marked as dash) post-stimulus time-intervals of the patients for the somatosensory oddball paradigm (Lindenbaum et al., 2023) and auditory paradigm (present study).

	Auditory		Somatosensory
Patient	nP3	P300	P300
01	—	—	—
02	—	—	☑
03	—	—	—
04	☑	—	☑
05	☑	☑	—
06	—	☑	☑
07	—	—	—
08	☑	—	☑
09	☑	—	—
10	—	—	—
11	☑	☑	☑
12	—	—	—
13	☑	—	☑
14	—	—	—

## 3 Results

### 3.1 Single-subject-aERP-analyses

**Figure 1:**
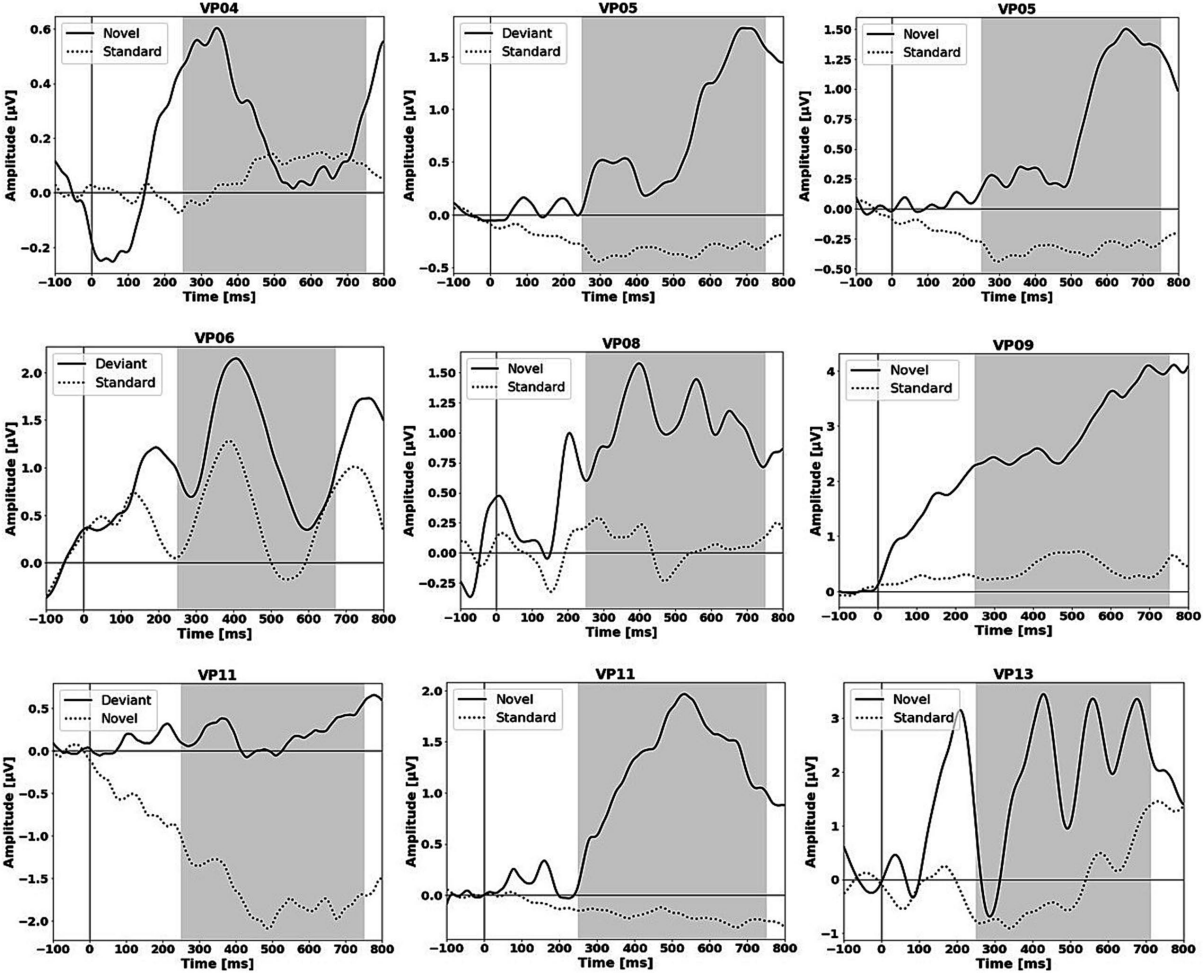
Grand-averaged aERP waveforms averaged across the significant electrode cluster. The gray background displays the significant time-interval.

**Figure 2:**
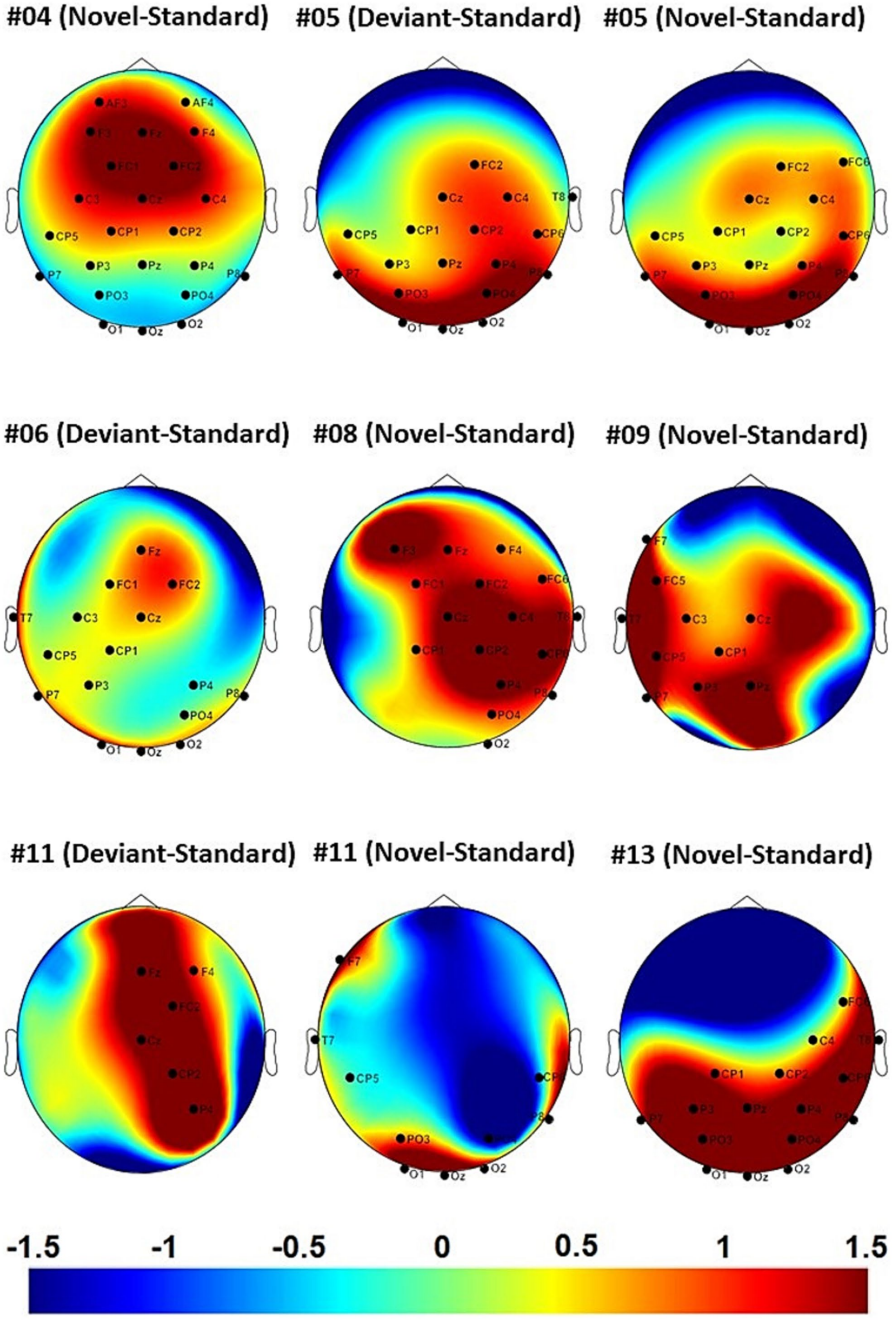
Topographic difference plots with significant electrode clusters in the significant post-stimulus time-interval. Depicted are the electrodes belonging to the significant electrode cluster.

For single-subject-aERP-analysis we defined two different time-intervals in which ERPs can typically be detected. An early interval for the N100 (0–250 ms) and a later interval for the P300 (250–750 ms). In the early N100 interval no significant difference between brain responses to the different stimuli was found among any of the comparisons or in any of the subjects. All results of the single-subject-aERP-analysis are listed in Table 5 and refer to the P300 interval. Time intervals with significant differences between neural responses to standard and deviant, or standard and novel or deviant and novel stimulation were detected in seven out of 14 subjects. The comparison between standard and novel stimuli most often led to significant differences in brain responses, suggesting the presence of a novelty P300. Two patients (05 and 11) had significant intervals for two comparisons: deviant vs. standard and novel vs. standard or deviant vs. novel stimuli. Patient 06 showed only significant differences in brain responses between the deviant and standard stimuli. The results of the auditory stimulation are depicted in Figure 1 and the significant clusters as well as the topographic difference plots are shown in Figure 2. The grey background in the waveform illustrations indicates the time interval where significant differences were observed (*p* < 0.05).

**Table 5:** Significant results of single-subject-aERP-analysis in the P300 time-interval (250–750 ms).

Patient	Comparison	Significant time interval (ms)	Cluster mass *p* Value	Ø AUC	Ø Max Amplitude (μV)	Ø Latency max. Amplitude (ms)
04	Novel—Standard	250–750	*p* = 0.014	87.53	2.37	471.61
05	Deviant—Standard	250–750	*p* < 0.001	593.37	4.52	553.36
	Novel—Standard	250–750	*p* < 0.001	543.94	4.25	545.36
06	Deviant—Standard	250–671	*p* = 0.019	292.08	4.18	417.16
08	Novel—Standard	250–750	*p* = 0.004	537.31	7.44	501.67
09	Novel—Standard	250–750	*p* = 0.027	1303.32	6.65	568.07
11	Novel—Standard	250–750	*p* = 0.013	777.21	3.44	518.57
	Deviant—Novel	250–750	*p* = 0.045	968.1	4.31	524.41
13	Novel—Standard	250–711	*p* = 0.042	1030.24	13.57	473.93

AUC, Maximum Amplitude and latency are averaged from the difference epochs of the significant time interval.

### 3.2 FFT-analysis

**Figure 3:**
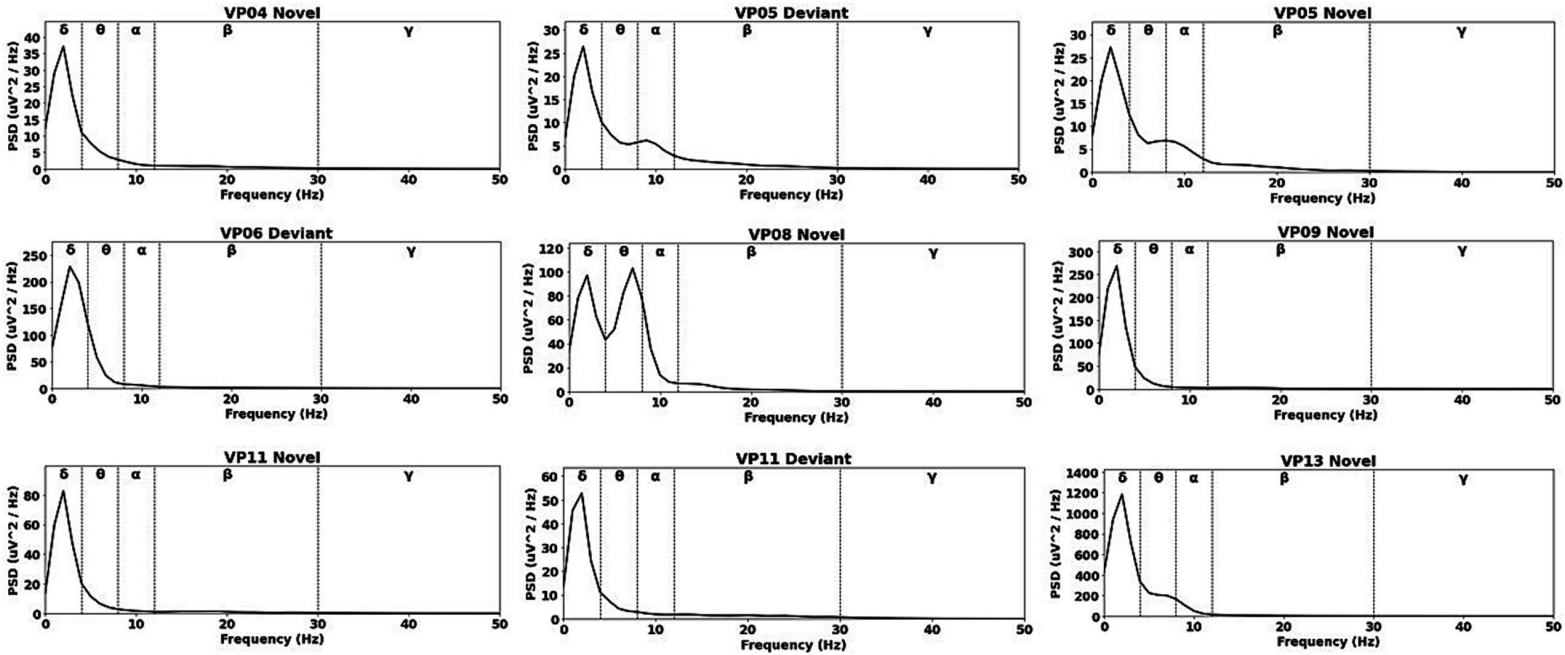
Averaged pre-stimulus frequency bands in significant electrode cluster.

Table 6 presents the averaged relative pre-stimulus frequency bands for deviant and novel trials among patients exhibiting significant post-stimulus aERP differences between standard and deviants, between standard and novels or between novels and deviants. The power spectral densities (PSDs) of the significant electrode cluster in the significant time interval 250–750 ms are shown in Figure 3. In the PSD illustrations, frequencies were restricted to 50 Hz due to the consistently low gamma power observed in all subjects, causing the PSD to nearly reach zero. The highest percentage of frequency bands was observed in the delta range, followed by the theta and alpha band for the majority of subjects. However, one subject (08) exhibited a higher percentage of theta than delta range.

**Table 6:** Results of FFT-analysis.

Patient	Comparison	Significant time interval (ms)	Ø Delta (%)	Ø Theta (%)	Ø Alpha (%)	Ø Beta (%)	Ø Gamma (%)
04	Novel—Standard	250–750	49.67	19.03	6.86	15.15	8.59
05	Deviant—Standard	250–750	48.15	16.65	12.75	16.32	5.62
	Novel—Standard	250–750	49.4	18.04	19.03	14.34	4.71
06	Deviant—Standard	250–671	65.93	20.96	4.77	5.89	2.25
08	Novel—Standard	250–750	35.91	39.29	14.93	9.15	0.67
09	Novel—Standard	250–750	71.38	12.39	3.16	9.05	3.68
11	Novel—Standard	250–750	54.59	11.32	5.65	18.04	9.59
	Deviant—Novel	250–750	54.32	13.19	5.56	15.66	10.2
13	Novel—Standard	250–711	60.13	26.27	9.93	3.04	0.58

Averaged relative pre-stimulus frequency bands for deviants or novels in significant electrode cluster of the significant post-stimulus time interval.

### 3.3 Correlation-analysis

The results of the correlation-analysis show a consistency in that pre-stimulus relative gamma power correlates negatively with post-stimulus variables in nearly all patients (see Table 7), although these correlations were not always significant. Among the seven subjects, who had significant post-stimulus time intervals in the single-subject-aERP-analysis, significant findings were observed in five cases. However, in patients 08 and 13 none of the correlations was significant. Patient 04 exhibited several moderate and weak correlations between pre-stimulus frequency components and post-stimulus variables, especially with the maximum amplitude. Beta and gamma activity correlated negatively and delta activity correlated positively with the maximum post-stimulus amplitude. Maximum amplitudes increased with decreasing beta and gamma activity and with increasing delta activity. And also, the AUC correlated positively with the delta activity. Patient 05 showed positive correlations with the beta and gamma band and the latency of the maximum amplitude of post-stimulus interval for the deviant and standard comparison and also a negative correlation between the delta band and latency of the maximum amplitude. Latencies of the maximum amplitudes increased with increasing beta and gamma band and decreasing delta band. Alpha, gamma and theta band showed a positive correlation with the maximum amplitude whereas the delta band correlated negatively with the maximum amplitude. Maximum amplitudes increased with increasing alpha, gamma and theta activity whereas the maximum amplitudes decreased with increasing delta activity. Furthermore, there was a negative correlation between pre-stimulus gamma activity and post-stimulus AUC, with the AUC increasing as gamma activity decreased. For the novel and standard comparison in patient 05, several weak correlations were found. Alpha and theta activity correlated positively with the latency of the maximum amplitude whereas delta correlated negatively with the latency of the maximum amplitude. Latencies of the maximum amplitudes were longer with increasing alpha and theta band and decreasing delta band. Post-stimulus AUC correlated negatively with the gamma band whereas the maximum amplitude correlated positively with the pre-stimulus gamma band. The AUC increased with decreasing gamma activity and the peak amplitudes were higher with increasing pre-stimulus gamma activity. Patient 06 had negative correlations between the gamma activity and post-stimulus AUC and maximum amplitude. AUC and peak amplitudes increased with decreasing gamma activity. Patient 09 showed positive correlations between pre-stimulus delta activity and post-stimulus AUC and the maximum amplitude and negative correlation between pre-stimulus theta band and AUC and the maximum amplitude. The maximum amplitudes and AUC were higher with increasing pre-stimulus delta activity and decreasing theta activity. Patient 11 had multiple weak and moderate negative correlations for the novel and standard comparison. Both, pre-stimulus beta and gamma activity correlated negatively with the post-stimulus AUC and maximum amplitude. The AUC and the peak amplitudes increased with decreasing beta and gamma activity.

**Table 7:** Spearman’s rho values of the correlation between pre-stimulus frequency band power and post-stimulus variables.

#04 Novel-Standard	δ	θ	α	β	γ
AUC	0.157*	−0.153	−0.075	−0.098	−0.144
Max. Amp.	0.301***	−0.078	−0.115	−0.300***	−0.405***
Lat. Max. Amp.	−0.088	0.120	−0.021	0.131	0.068

#05 Deviant-Standard	δ	θ	α	β	γ
AUC	0.049	0.073	−0.075	0.044	−0.155*
Max. Amp.	−0.259***	0.242**	0.165*	0.098	0.242**
Lat. Max. Amp.	−0.220**	0.082	0.134	0.212**	0.232**

#05 Novel-Standard	δ	θ	α	β	γ
AUC	0.082	0.034	−0.089	0.031	−0.183*
Max. Amp.	−0.082	0.115	0.090	−0.029	0.151*
Lat. Max. Amp.	−0.178*	0.222**	0.194*	0.102	0.042

#06 Deviant-Standard	δ	θ	α	β	γ
AUC	−0.030	0.131	−0.051	−0.093	−0.190*
Max. Amp.	0.035	0.068	−0.082	−0.109	−0.208*
Lat. Max. Amp.	−0.047	0.089	−0.090	−0.033	−0.040

#08 Novel-Standard	δ	θ	α	β	γ
AUC	0.023	−0.041	0.012	−0.098	−0.095
Max. Amp.	−0.028	0.054	−0.035	−0.089	−0.113
Lat. Max. Amp.	0.005	−0.007	−0.067	−0.028	−0.004

#09 Novel-Standard	δ	θ	α	β	γ
AUC	0.256*	−0.321**	−0.092	−0.153	−0.176
Max. Amp.	0.243*	−0.312**	−0.102	−0.140	−0.171
Lat. Max. Amp.	−0.120	−0.045	0.173	0.215	0.156

#11 Novel-Standard	δ	θ	α	β	γ
AUC	0.216	0.229	−0.143	−0.354*	−0.456***
Max. Amp.	0.193	0.168	−0.113	−0.306*	−0.419**
Lat. Max. Amp.	−0.081	−0.033	−0.066	0.097	0.111

#11 Deviant-Novel	δ	θ	α	β	γ
AUC	−0.009	−0.110	0.000	0.079	0.017
Max. Amp.	−0.046	−0.081	−0.008	0.098	0.041
Lat. Max. Amp.	0.047	0.069	0.036	−0.087	−0.160

#13 Novel-Standard	δ	θ	α	β	γ
AUC	0.016	0.044	−0.147	−0.117	0.113
Max. Amp.	0.162	−0.108	−0.239	−0.186	0.030
Lat. Max. Amp.	−0.071	0.064	0.089	0.023	0.070

Significance levels are denoted as follows: *p < 0.05, **p < 0.01, *** *p* < 0.001. Significant correlations are indicated in bold, Bonferroni corrected significant correlations are underlined.

### 3.4 Additional analyses

**Figure 4:**
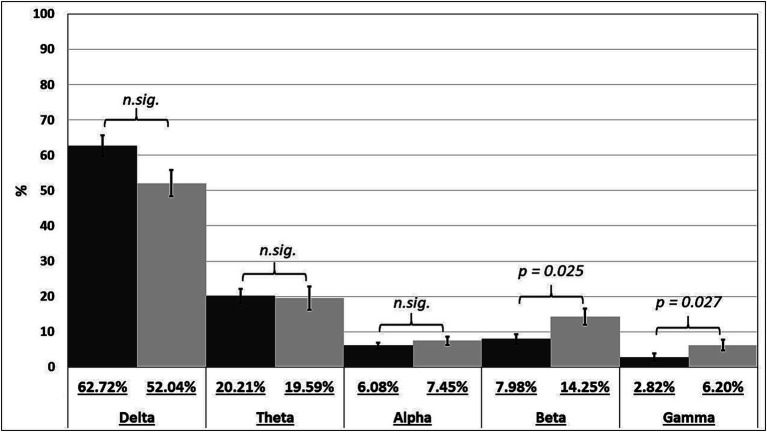
Averaged relative power of pre-stimulus frequency bands of patients with no significant differences between the stimuli in the post-stimulus interval (dark gray) and patients with significant differences between the stimuli in the post-stimulus interval (light gray).

Further analysis showed that there are significant differences between the amount of relative beta and gamma activity when comparing the pre-stimulus frequencies between the significant and insignificant post-stimulus intervals. The post-stimulus intervals of 9 data sets (deviant/novel versus standard) were significant. Results showed that the relative power of the beta (*U* = 40.0, *p* = 0.025) and gamma band (*U* = 40.0, *p* = 0.027) were higher in the pre-stimulus interval when post-stimulus intervals where significantly different between the stimuli in contrast to post-stimulus intervals which were not significantly different between the stimuli (see Figure 4). Also, a trend towards significance for the relative delta power was observed (*U* = 46.0, *p* = 0.054).

Table 4 shows the overview of the significant and insignificant post-stimulus intervals from our previous somatosensory study (Lindenbaum et al., 2023) and the present study. Six patients did not yield significant findings in the somatosensory nor in the auditory paradigm. Six patients showed significant post-stimulus intervals in both modalities. Two patients did not yield significant time-intervals in the somatosensory paradigm but they did in the auditory one. Conversely, one patient displayed significant time intervals in the somatosensory study but not in the auditory study.

## 4 Discussion

In this study we explored if pre-stimulus oscillations have an effect on post-stimulus aERP indicators of target and nontarget stimuli differentiation in patients with DoC. A total of 14 patients with DoC took part. Among them, seven individuals (50%) exhibited notable distinctions in brain responses to deviant and novel stimulation versus standard stimulation and also to deviant versus novel stimulation within specific post-stimulus time intervals and electrode clusters. Out of these, five subjects (35.71%) demonstrated several statistically significant correlations between pre-stimulus frequency bands and post-stimulus variables. Four of them were in the MCS state and one patient was between the stages of UWS and MCS.

### 4.1 Single-subject-aERP-analysis

#### 4.1.1 N100

No significant differences were found in the N100 time-interval in any of the patients. This outcome does not necessarily signify that no N100 could be found in the patient’s brain responses but rather indicates that the differences between the N100 to target, nontarget and standard were not significant. In general, the occurrence of N100 is notably more frequent compared to the P300 in patients with DoC (Kotchoubey et al., 2005; Real et al., 2013). Moreover, the N100 as well as other ERPs are known to differ in latency in patients in DoC with a significant delay compared to healthy participants (Glass et al., 1998) but nevertheless our chosen time-interval of 250 ms should have covered the N100.

#### 4.1.2 P300

Seven patients showed no significant differences between brain responses to either deviant or novel stimulation versus standard stimulation nor to deviant versus novel stimulation within the P300 post-stimulus time interval (see Table 5). In one patient (07) artifact free trials were likely insufficient for the analysis (see Table 2) due to excessive motion and movement of the patient. It is known that the P300 is less common in people with DoC than in healthy individuals (Gott et al., 1991; Kane et al., 2000; Signorino et al., 1997). The lack of significant findings in these patients does not automatically imply a complete absence of noteworthy distinctions in their brain responses to specific stimuli. Fatigue or variations in arousal levels might have contributed to the inability to observe significant differences in several patients. Reduced activity due to arousal fluctuations could lead to the patient’s inability to stay attentive and differentiate between the stimuli (Neumann and Kotchoubey, 2004). Compared with the somatosensory findings, there is also the possibility that specific lesions impair P300 generation in one particular modality. This highlights the need for multi-modal assessments.

##### 4.1.2.1 Post-stimulus differentiation

Additional examination revealed notable variances in the levels of relative beta and gamma activity when comparing pre-stimulus frequencies between significant and insignificant post-stimulus intervals (see Figure 4). Although the relative power of beta and gamma were significant higher in the pre-stimulus interval for the significant data sets the correlation analysis showed a negative correlation between these frequency bands and post-stimulus variables in most cases. High frequency activity (e.g., in the beta and gamma band) is associated with wakefulness, selective attention (Müller and Keil, 2004) and active cortical processes like memory (Gruber et al., 2004). The fact that significant post-stimulus data sets are accompanied by significantly higher relative band power of the gamma and beta band in the pre-stimulus interval could be due to attention or memory processes and also to wakefulness of the patient and therefor a significant difference between the deviant/novel and standard stimuli was detectable. Nevertheless, this finding is at odds with the results of the correlation analysis. A further explanation for this is maybe that the overall relationship follows a U-shaped pattern, suggesting that while it is generally advantageous to possess higher pre-stimulus beta and gamma for perceptual differentiation, there may exist an optimal level. The group exhibiting post-stimulus differentiation demonstrates greater high-frequency activity compared to those without it. However, individual instances may show a negative correlation, as excessive high-frequency activity can also be detrimental.

##### 4.1.2.2 Effects of stimulation modality

In addition, the stimulation paradigm could also have contributed to the insignificant results in the aERP-analysis. In our previous study (Lindenbaum et al., 2023) we conducted a somatosensory oddball study with the same patients as used in this study. On average, as many patients showed auditory as somatosensory responses but the distribution differed: Patient 09 and 05 did not exhibit significant time intervals in the somatosensory paradigm; however, they did in the auditory paradigm. In contrast, Patient 02 showed significant time intervals in the somatosensory study but not in the auditory study. (see Table 4). This finding highlights the significance of employing multi-sensory paradigms in neuroimaging studies of patients with DoC and is consistent with other studies like Annen et al. (2020) and Rousseau et al. (2008). Annen et al. (2020) used an EEG-based BCI with auditory and somatosensory stimulation to evaluate the response of DoC patients to a P300 paradigm. They examined the datasets of 40 patients. Seven of them showed only a ‘differentiated response’ in the auditory paradigm, 11 patients showed only a ‘differentiated response’ in the somatosensory paradigm, six of them showed a ‘differentiated response’ in both paradigms and 16 patients did not show a ‘differentiated response’ to any of the two paradigms.

Rousseau et al. (2008) stimulated four patients in the persistent vegetative state with three different sensory paradigms (auditory, somatosensory and visual). The results were very variable with one patient showing no evoked potentials (EPs) to the visual paradigm but to the other two paradigms, one patient showed only EPs to the visual paradigm, the other patient showed no responses to the auditory paradigm but to the somatosensory and visual paradigm and the last patient had EPs to the somatosensory stimulation, abnormal EPs to the visual stimulation and no responses to the auditory presentation of stimuli. These results indicate that the brain responses to stimulation paradigms are individually different in patients in DoC and that not all modalities are effective and useful to elicit ERP differences in all of the patients. Given the significance of the matter, it is crucial to utilize a multimodal assessment approach for DoC patients to address individual impairments effectively. This strategy enhances the discovery of potential latent abilities and facilitates the identification of the most suitable sensory modality for each patient, as emphasized by Annen et al. (2020).

##### 4.1.2.3 nP3 vs. P3b

Only one patient (05) showed significant time-intervals in both comparisons, deviant vs. standard and novel vs. standard, with similar waveforms in the post-stimulus P300 interval but with descriptively lower amplitude maxima for the novel stimuli (see Table 5 and Figure 1). It is known that the amplitude of the P300 is significantly higher when MCS patients follow instructions to actively attend to or count a certain stimulus compared to the passive condition when the patient was instructed to just listen to the sounds (Schnakers et al., 2008; Risetti et al., 2013). The same applies to healthy subjects, with P300 amplitudes expected to be larger for task-relevant conditions compared to passive processing (Polich, 1998). Although the amplitudes for the deviant stimuli are descriptively higher than the amplitudes of the novel stimuli, it does not necessarily imply that this patient was capable to follow the instruction to actively count the occurrence of the deviants. Furthermore, the statistical analysis demonstrated no significant differences (*p* = 0.322) between the deviant and novel amplitudes, preventing us from making any assumptions in this regard. Nevertheless, the comparison between standard and novel stimuli most often led to significant differences in brain responses despite the active condition to count the deviant. The novelty P300 offers valuable insights into the functional status of a patient’s cortex without necessitating their direct engagement or active attending (Morlet and Fischer, 2014) and reflects a strongly-automatic process (Muller-Gass et al., 2007). There are multiple studies in which the nP3 component was found in the majority of the patient sample group in response to passive listening to, e.g., the subject’s own name (SON) (Fischer et al., 2010; Risetti et al., 2013; Perrin et al., 2006). Also, our results indicate that the nP3 could be an automatic process because the brain responses to the actively counted deviants did not significantly differ from the brain response to standard stimuli in some of the patients but the differences between the novel and standard stimuli were significant in the specific P300 time-interval. Considering the extent of their neurological and clinical impairment it could be the case that the patients were not aware of the stimuli (Chennu and Bekinschtein, 2012). The P300 is linked to conscious perception of outstanding stimuli (Sutton et al., 1965). The observed disparities between standard and novel stimuli during the post-stimulus interval may be attributed to the phenomenon wherein certain patients can consciously perceive substantial auditory deviations or changes in their surroundings but their inability to sustain attention on stimuli may account for the absence of a P3b response.

#### 4.1.3 Summary

Identifying the presence or absence of post-stimulus aERPs can be challenging in certain patients, especially through visual inspection alone. Patients 06, 08 and 13 exhibited highly irregular waveforms in the post-stimulus interval (see Figure 1). However, the statistical analyses demonstrated significant differences in brain responses to deviant and novel stimuli in contrast to standard stimuli within specific time intervals and electrode clusters. Conversely, the aERP curves of patient 04 displayed distinct ERP components (N100 and novelty P300) in response to novel stimuli. Regarding the topographies (see Figure 2), it can be noted that certain ones are typical, while others are atypical. Patient 04 shows a typically fronto-central distribution of the nP3 to novel stimuli and patient 05 shows an almost typically centro-parietal distributed P3b to deviant stimuli but also with an occipital shift. In addition, patient 05 shows almost the same distribution for the nP3 with a quite similar significant electrode cluster. The topographies of the other patients are rather atypical. However, in individuals with extensive cortical lesions, an abnormal topography is expected and not uncommon (Kotchoubey et al., 2005).

### 4.2 FFT

In line with previous findings (Claassen et al., 2004; Fellinger et al., 2011; Nagata et al., 1989) our results showed that the delta band power is predominant in DoC-patients. The pre-stimulus relative power of six out of the seven patients with significant post-stimulus effects consisted predominantly of the delta frequency band (see Table 6 and Figure 3). Despite the fact that delta was dominant in the pre-stimulus power spectrum in these patients the analysis of their post-stimulus interval showed significant differences between the stimuli. Patient 08 showed higher pre-stimulus relative theta than delta power.

In healthy awake adults, a symmetrical alpha rhythm is observed at posterior electrodes during periods of rest with an overlaid beta rhythm when they are attentive to their surroundings (Lehembre et al., 2012a). The theta rhythm becomes prominent when an individual is tired, and the delta rhythm predominates during deep sleep states (Lehembre et al., 2012a). In DoC-patients there are significant abnormalities in the power of frequency bands, characterized by reduced power in the alpha band and increased power in the delta band which reflects a low consciousness level (Sebastiano et al., 2015). Additionally, differences in power between patients in UWS and those in MCS are also observed (Lehembre et al., 2012b). Furthermore, due to diffusely slow activity, many abnormal patterns can be observed, like continuous focal polymorphic delta rhythm and epileptiform activity, detected in areas of brain damage (Brenner, 2005).

### 4.3 Correlation-analysis

Multiple studies examined the connection between pre-stimulus oscillations and post-stimulus outcomes following stimulation in healthy individuals. Here, specifically, activity occurring before the stimulus, particularly in the alpha band, has the potential to influence both early and late post-stimulus auditory ERPs (Barry et al., 2000; Haig and Gordon, 1998; Intriligator and Polich, 1995; Jasiukaitis and Hakerem, 1988) and visually-evoked ERPs (Ergenoglu et al., 2004; Mathewson et al., 2009; Roberts et al., 2014) concerning their amplitude peaks and the latency to reach these peaks.

Significant correlations were identified between the relative power of frequency components during the pre-stimulus interval and post-stimulus variables of aERPs in five out of the seven patients who exhibited significant differences between neural responses to deviant or novel stimuli and standard stimuli (see Table 7). Patient 08 and 13 showed no significant correlations between pre-stimulus frequencies and post-stimulus variables.

Patient 04 had multiple correlations for the comparison between novel and standard stimuli. The maximum amplitude and AUC of post-stimulus interval increases with higher pre-stimulus relative delta power and the amplitudes maximum decreases with higher pre-stimulus beta and gamma power. De Blasio and Barry (2013a) conducted an auditory Go/NoGo study in which healthy subjects had to perform a button-press response (Go-Task) to a specific stimulus investigating the influence of pre-stimulus low-frequencies on post-stimulus ERPs. Their results showed that post-stimulus P300 amplitudes are modulated by high pre-stimulus delta, with post-stimulus amplitudes being more positive with high pre-stimulus delta, but this modulation did not differ between the stimulus conditions. Our results of patient 04 and patient 06 are in line with this outcome. Usually P300 amplitudes vary across different stimulus conditions. Since no differences between the P300 amplitudes in the Go vs. NoGo task were found, De Blasio and Barry (2013a) assumed that pre-stimulus delta activity has minimal or no effect on endogenous processing. The results of our study are not quite in line with this outcome. The nP3 is believed to index exogenous attention and is therefore task-irrelevant, whereas the P3b is linked to “top-down“-processes reflecting endogenous attention. The patients showed only positive correlations, if significant, between pre-stimulus delta power and post-stimulus AUC or maximum amplitude in the novel vs. standard comparison and negative correlations in the deviant vs. standard comparison. Still, it is unclear if the patients were really aware of the deviant stimuli. The negative correlation between pre-stimulus gamma and post-stimulus maximum amplitude in patient 04, patient 06 and patient 11 is converse to what is typically found in healthy subjects. Healthy subjects have been found to show positive correlations between pre-stimulus gamma power and post-stimulus P300 amplitudes (Reinhart et al., 2011) with post-stimulus P300 amplitudes being more positive the higher the pre-stimulus gamma power. According to the findings of Gómez et al. (2004), reduced pre-target gamma power could enhance the processing of relevant task-related information. Despite the fact that the novel stimulus was not task-relevant and the nP3 is linked to “bottom-up”-processes, the pre-stimulus low gamma activity correlated with better detection of the novel stimulus in the sequence of standard and deviant stimuli leading to higher post-stimulus amplitudes in these patients.

In patient 04 and 11 the pre-stimulus beta power correlated negatively with the post-stimulus maximum amplitude and also with the AUC in patient 11 in the novel vs. standard comparison. Increased pre-stimulus beta activity led to lower nP3 amplitudes and a lower AUC. Van Ede et al. (2010) conducted a tactile MEG-experiment to investigate if the expectation of a tactile stimulus involves a pre-stimulus modulation of oscillations in the somatosensory cortex. They assumed that beta oscillations indicate a brain state in which the efficiency of neuronal processing is reduced and that the suppression of beta oscillations prepares the system for upcoming event-processing. Although we used an auditory paradigm in this study and did not focus on a specific brain region for the oscillation analysis it seems that this outcome is in line with our finding showing that the processing of the novel stimulus is deteriorated leading to lower post-stimulus amplitudes when beta is enhanced in the pre-stimulus time-interval. However, there are also controversial results in previous research focusing on beta oscillations in the pre-stimulus time-interval. In healthy individuals, pre-stimulus beta activity has been observed to normally influence earlier event-related potentials and alpha activity appears to modulate post-stimulus P300 in both auditory and visual paradigms (De Blasio and Barry, 2013b). Taking this together the observed result should be approached with caution and should be explored in future research.

Patient 09 showed a negative correlation between pre-stimulus theta power and post-stimulus AUC and maximum amplitude with AUC and amplitudes being lower with increased pre-stimulus theta power. This outcome is at odds with the observation made in healthy people. Multiple studies reported positive correlations between pre-stimulus theta activity and post-stimulus P300 (Başar et al., 1984; De Blasio and Barry, 2013a; Yordanova and Kolev, 1998; Lazzaro et al., 2001) in healthy people. It is challenging to interpret our result but it seems that, at least in this patient, low theta activity in the pre-stimulus interval contributes to the processing of novel stimuli producing higher nP3 amplitudes and AUCs.

Patient 05 had multiple moderate and weak correlations in the deviant vs. standard and also novel vs. standard comparison. This patient had, in contrast to the other patients, a relatively normal pre-stimulus frequency spectrum. While, as a group, the other patients showed similar correlation patterns, this patient exhibited different correlations from the other patients but similar correlations to those found in healthy subjects. The patient showed weak correlations between pre-stimulus alpha power and post stimulus paradigms. It has been proposed that pre-stimulus alpha power increases the likelihood of becoming consciously aware of a post-stimulus visual stimulus but does not influence visual sensitivity when making decisions about specific features of the stimulus (Benwell et al., 2017; Benwell et al., 2022). Extending this finding to the auditory modality, this patient may have the capacity to consciously perceive auditory stimuli due to pre-stimulus alpha activity, while their sensitivity to discriminate specific auditory features of previously presented standard stimuli remains unaffected.

Considering the clinical state of the patients, it is not clear if the patients understood the instructions of the auditory paradigm. Results showed that the presentation of the novel stimulus led to significant differentiation from the standard stimulus more frequently than did the target stimulus. This may be attributed to the fact that the nP3 is linked to a more automatic response than the P3b (Segalowitz et al., 2001). Nevertheless, pre-stimulus relative frequency power, especially the gamma band, correlated with post-stimulus variables, also in the actively counting condition (target) but “bottom-up”-processes are more reliable in DoC patients at least in our study.

## 5 Limitations and conclusion

Just seven out of 14 patients showed significant differences in post-stimulus brain responses to the different auditory stimuli. This outcome shows that not all patients had post-stimulus ERPs and is consistent with other studies (e.g., Lindenbaum et al., 2023; Schoenle and Witzke, 2004; Kotchoubey et al., 2005). Our results also indicate that in subjects with DoC the pre-stimulus EEG activity can correlate to post-stimulus aERPs. Multiple correlations, some atypical and some in line with healthy participants were found. The lack of significant findings in the remaining seven patients does not necessarily imply that there are no significant differences in their brain responses to specific stimuli, it rather highlights the importance of employing multi-sensory paradigms in neuroimaging studies of patients with DoC (see also Rousseau et al., 2008). In five out of the seven subjects with significant post-stimulus differences, we were then also able to find significant correlations between relative pre-stimulus frequency bands and post-stimulus aERP variables. Considering that we computed 15 correlations for each patient, it is natural to anticipate some chance correlations but since the strict Bonferroni corrected significance level is 0.003, several of the correlations would still be significant. However, due to the overall alterations in EEG spectra among DoC patients, it is likely that there are atypical yet still functional brain dynamics.

Our findings indicate that pre-stimulus oscillations are related to post-stimulus sensory and cognitive processing, although these effects vary highly from one individual to another. Identifying these individual relationships could aid in determining optimal stimulation windows for patients with DoC, potentially improving the chances of effective stimulation, thereby supporting patients on their path to recovery. Overall, our study contains a relatively small sample size. However, here, we present a relatively detailed analysis of individual cases that should be informative and inspire further research. This level of detail is likely impossible in larger studies. Therefore, present results could inform larger, potentially also multi-center studies.

## Data Availability

The raw data supporting the conclusions of this article will be made available by the authors, without undue reservation.

## Footnotes

^1^ http://www.biosemi.com
